# Spatial perspective taking is impaired in spinocerebellar ataxias and Friedreich ataxia

**DOI:** 10.1038/s41598-025-16302-z

**Published:** 2025-08-24

**Authors:** Simona Karamazovova, Martina Laczó, Veronika Matuskova, Natalie Svecova, Lucie Stovickova, Zuzana Blichova, Jaroslava Paulasova Schwabova, Michaela Kuzmiak, Jan Laczó, Martin Vyhnalek

**Affiliations:** 1 Center of Hereditary Ataxias, Department of Neurology, Second Faculty of Medicine, Charles University and Motol University Hospital, Prague, Czech Republic; 2 Department of Clinical Psychology, Motol University Hospital, Prague, Czech Republic; 3 Center of Hereditary Ataxias, Department of Pediatric Neurology, Second Faculty of Medicine, Charles University and Motol University Hospital, Prague, Czech Republic

**Keywords:** Spinocerebellar ataxia, Friedreich ataxia, Cerebellum, Spatial navigation, Spatial perspective taking, Cognition, Cognitive neuroscience, Neurodegeneration

## Abstract

Spinocerebellar ataxias (SCA) are rare neurodegenerative diseases affecting the cerebellum and its connections, leading to progressive motor disability and cognitive impairment as part of the cerebellar cognitive affective syndrome. Spatial navigation, cognitive function important for everyday movement, relies on spatial perspective taking—the ability to imagine the environment from different viewpoints. While animal and neuroimaging studies suggest a crucial role of the cerebellum in spatial navigation, research on patients with cerebellar disorders is lacking. This study aimed to investigate perspective taking in patients with SCA and Friedreich ataxia (FRDA) using two tests. The Perspective-Taking/Spatial Orientation Test (PTSOT) was administered to 30 SCA patients, 30 FRDA patients, and 34 healthy controls (HC). In addition, SCA and HC completed the Directional-approach Task and a comprehensive neuropsychological assessment. SCA patients performed significantly worse than HC on both perspective taking tests. FRDA patients performed better than SCA and differed from HC only in a subset of PTSOT measures. Perspective taking performance in SCA was associated with global cognition and multiple cognitive domains but not with cerebellar motor impairment. These findings are of potential clinical relevance, as spatial navigation deficits are known to negatively affect the mobility and independence of the affected individuals. Our findings expand the understanding of cognitive impairments in cerebellar diseases, adding spatial navigation to the spectrum of the cerebellar cognitive affective syndrome.

**Supplementary Information:**

The online version contains supplementary material available at 10.1038/s41598-025-16302-z.

## Introduction

Spinocerebellar ataxias (SCA) are a large group of rare, debilitating neurodegenerative diseases affecting the cerebellum and its connections, predominantly manifesting as a cerebellar syndrome. The SCA are heterogeneous in terms of mode of inheritance, brain regions involved, and clinical features. The majority of autosomal dominant and many recessive SCA primarily affect the cerebellar cortex, in contrast to Friedreich ataxia (FRDA), the most common autosomal recessive ataxia, which predominantly affects the spinal cord and deep cerebellar nuclei^1^.

In ataxia patients, cerebellar motor symptoms, such as progressive gait and limb incoordination or dysarthria, are often accompanied by cerebellar cognitive affective syndrome (CCAS)**,** a condition characterized by a spectrum of cognitive and neuropsychiatric features. Cognitive impairment primarily involves deficits in executive, language, and visuospatial functions and is likely attributed to the disruption of cerebellar connections with the cerebral cortex, particularly the frontal, parietal, and temporal regions^2–4^. These symptoms have been described in SCA, and to a lesser extent in FRDA, presumably due to a less pronounced cerebellar grey matter atrophy^5,6^. Several studies have demonstrated a dissociation between the CCAS and the motor disability, although this distinction has been challenged by others, particularly in SCA2 and SCA3^7–12^.

Beyond these well-documented cognitive deficits**,** spatial navigation represents another important but less explored domain with evidence of cerebellar involvement^13–15^. Spatial navigation is a complex cognitive process critical for meaningful movement in the environment and finding the way from one place to another in daily life. One of the key components of spatial navigation is perspective taking, which is the ability to imagine the environment from a different viewpoint. This ability is essential for recognizing familiar locations from new perspectives, for example, when approaching an intersection from an unfamiliar direction. It is primarily associated with function of the parietal and temporal cortex^16,17^.

Spatial navigation impairment is well-documented in neurodegenerative diseases and reflects specific structural brain changes in these disorders. The most thorough research has been conducted in Alzheimer disease, where severe global spatial navigation deficits have been reported, including impairments in perspective taking^18^, associated with atrophy of the medial temporal lobe and parietal cortex^19^. Spatial navigation deficits were also reported in Huntington and Parkinson diseases, where they have been associated with striatal and cortical degeneration^20–23^.

Emerging evidence from animal studies and functional neuroimaging human studies highlights a major role of the cerebellum in spatial navigation**.** Cerebello-hippocampal interactions have been shown to contribute to successful navigation, trajectory optimization, and cognitive map formation in studies using functional neuroimaging in healthy humans, as well as in experiments with cerebellar mutant mouse models in spatial paradigms^14,15^. Furthermore, functional MRI studies revealed cerebellar activation during perspective taking tasks in healthy individuals^24,25^.

Spatial navigation is a multifaceted ability that depends on the integration of several cognitive functions, particularly visuospatial, and executive functions, as well as memory^26^. There is evidence for impaired visuospatial processing in cerebellar patients, including deficits in mental rotation tasks, which are closely related to perspective taking^27^. These deficits were observed particularly in patients with left-sided lesions in the posterior lobe of the cerebellum and are thought to reflect the disrupted connectivity between this region and the cortical association areas, particularly in the parietal lobe^28^.

Despite the growing evidence for the role of the cerebellum in spatial navigation, including perspective taking, studies on patients with degenerative cerebellar disorders are lacking. Investigating these impairments could provide clinically valuable insights, as spatial navigation deficits are known to significantly hinder daily functioning and quality of life^29^. In patients with cerebellar diseases, such deficits may further hamper their mobility and independence in the environment, compounding the effects of motor impairment.

Spatial navigation tests have a great potential to detect spatial navigation impairment, however, they might not be widely available in clinical setting. Spatial navigation questionnaires might offer another possibility to assess spatial navigation skills in everyday life^30^. Previous studies showed that self-reported spatial navigation questionnaires might detect navigation deficit in early stages of neurodegenerative disease, specifically in Alzheimer disease^31,32^. Until now, no studies have analyzed spatial navigation performance or self-reported spatial navigation abilities in patients with SCA.

This study focuses on spatial perspective taking, which is a key component of spatial navigation, in patients with degenerative ataxias and represents the first investigation of spatial navigation aspects in individuals with cerebellar disorders. We aimed to (1) compare perspective taking abilities in patients with SCA and FRDA to those of healthy controls (HC), (2) examine the relationship between perspective taking, general cognition and specific cognitive domains, (3) investigate the relationship between the perspective taking impairment and the cerebellar motor impairment in ataxia patients, 4) assess self-reported spatial navigation abilities in patients with SCA and explore their relationship with the performance on perspective taking tests.

Given the available information on the role of the cerebellum in spatial navigation, we hypothesized that ataxia patients would perform significantly worse on the perspective taking tests compared to HC, and that the results would correlate with general cognition, executive and visuospatial functions, but not with cerebellar motor impairment. Given the less pronounced cerebellar cortical atrophy in FRDA, we anticipated that SCA patients would show greater deficits than FRDA patients. Furthermore, we hypothesized that ataxia patients would report greater spatial navigation difficulties than HC and that these self-reported difficulties would be associated with poorer performance on perspective taking tests.

## Methods

### Participants

We conducted a single-center cross-sectional study with 94 participants. Sixty patients with degenerative cerebellar ataxias were recruited from the Center of Hereditary Ataxias, Prague, Czech Republic. This group included 24 genetically confirmed SCA patients (3 SCA1, 11 SCA2, 1 SCA3, 1 SCA6, 1 SCA8, 1 SCA14, 3 SCA17, 1 SCA21, 1 SCA27B, 1 SCA28), 2 progressive ataxias due to CACNA1A point mutation NM_001127221.1 (CACNA1A):c.1748G>A (p.Arg583Gln), two patients with CANVAS, one patient with SPG7, one patient with ataxia with oculomotor apraxia type 2, and 30 FRDA patients. All ataxia participants were clinically symptomatic, over 18 years of age, and native Czech speakers^33^. For the purpose of this study, patients were divided into two groups: the FRDA group and the SCA group, which included all other patients with degenerative cerebellar ataxias.

The HC group was recruited from among the acquaintances of the hospital staff, healthy relatives of the patients, or via social media, and comprised 34 volunteers with no history of neurological or other significant medical conditions. All HC participants reported no subjective cognitive concerns and scored within normal limits on the neuropsychological battery.

Individuals with a history of other neurological diseases potentially influencing cognition (stroke, craniocerebral trauma, other neurodegenerative disorders, etc.), non-compensated general medical or psychiatric conditions, or a history of substance abuse were not included. To avoid the influence of visual disturbance, all participants underwent examination with the Snellen charts and had normal or corrected-to-normal vision. All participants gave their written informed consent, and the study was conducted with the approval of the local ethics committee of Motol University Hospital and in accordance with the Declaration of Helsinki.

### Procedures

The ataxia (SCA and FRDA) patients underwent neurological examination, which included the Scale for the Assessment and Rating of Ataxia (SARA)^33^. Additionally, self-reported Activities of Daily Living Scale (FA-ADL)**,** a questionnaire specifically tailored to evaluate difficulties in mainly motor everyday activities commonly affected in ataxia patients, was administered. These activities include speech, gait, personal hygiene, and other tasks requiring fine motor control and coordination. The FA-ADL scale assesses both the level of difficulty patients experience in performing these tasks and the extent of assistance they require to complete them^34^.

SCA patients and HC underwent a research protocol focused on spatial navigation, which included a complex neuropsychological examination, two tests of spatial perspective taking (Perspective Taking/Spatial Orientation Test (PTSOT)^35^ and the Directional-approach Task from the Navigation Test Suite^36^) and also the Santa Barbara Sense of Direction Scale (SBSOD) to assess self-reported spatial navigation abilities^37^.

FRDA patients only completed the PTSOT and the Montreal Cognitive Assessment (MoCA) to assess cognitive functions^38^. The Mini-Mental State Examination (MMSE) scores were derived from the MoCA results for standardized comparison^39^. The PTSOT test was administered to FRDA patients during their yearly EFACTS visit.

### Neuropsychological assessment

The neuropsychological battery included the MMSE^40^ as a screening of global cognitive function and the following tests to assess five cognitive domains: (1) memory by the Rey Auditory Verbal Learning Test and Logical Memory from the Uniform Data Set; delayed recall)^41,42^; (2) executive function by the Trail Making Test B (TMT B), phonemic verbal fluency—letters K, P and S, Wisconsin Card Sorting Test (WCST)—perseverative errors, and the Stroop Color and Word test—color-word condition^43–46^; (3) language by the Boston Naming Test 30-item version and category verbal fluency—animals and vegetables^42^; (4) attention and working memory by the Trail Making Test A (TMT A), and Digit Span forward and backward from the WAIS-III^47,48^; and (5) visuospatial function by the Judgement of Line Orientation^49^. Composite Z-scores for all cognitive domains were calculated as the mean of relevant test Z-scores, with WCST, TMT A and TMT B scores reversed prior to transformation. Depressive symptomatology was assessed by the Beck Depression Inventory (BDI) questionnaire^50^.

### Perspective-taking/spatial orientation test

**Fig. 1:**
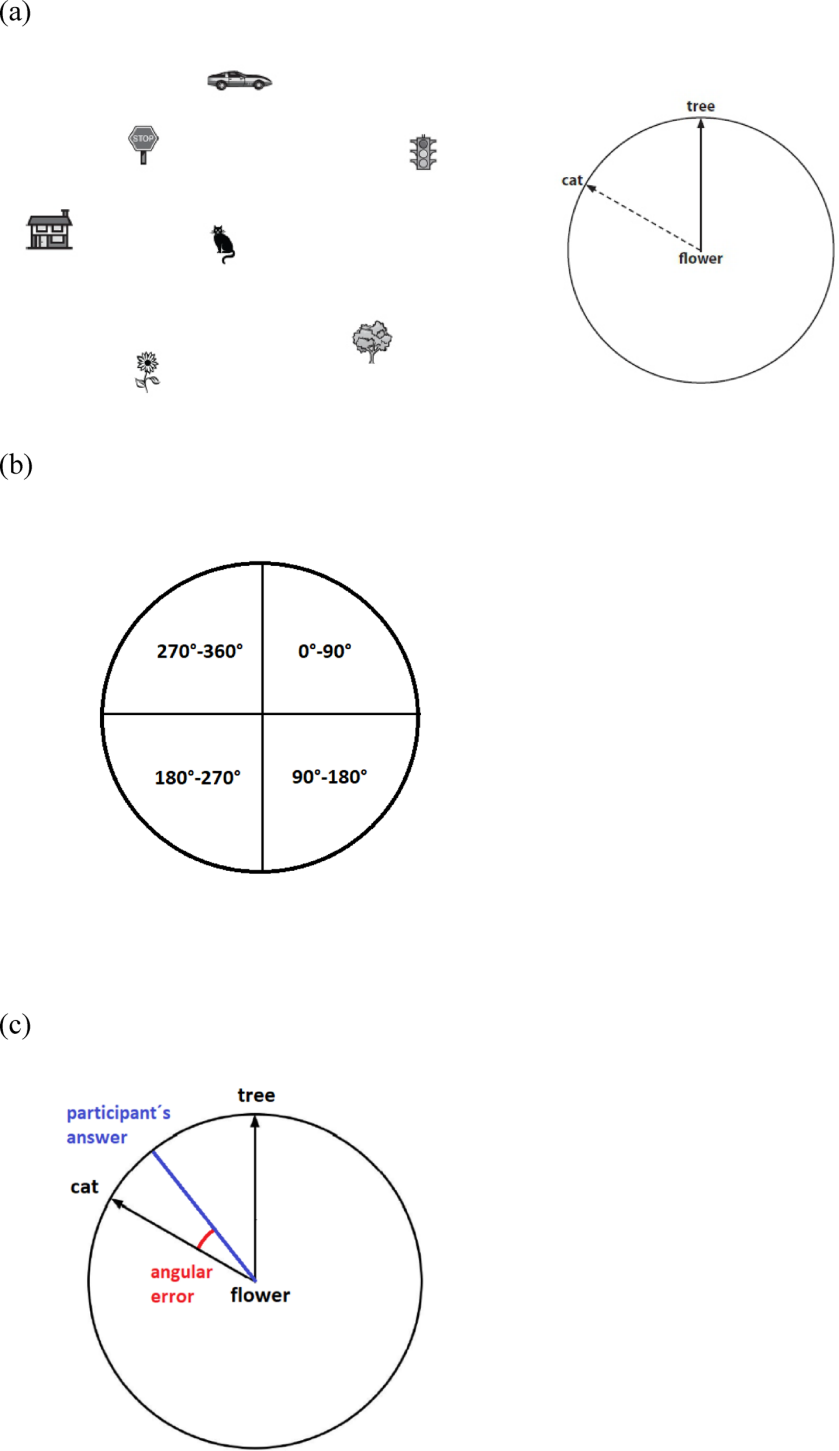
(**a**) Example of the PTSOT task—Participants are asked to imagine standing at one object (e.g., flower) and facing another (e.g. tree). Their task is to indicate the direction to a third object (e.g., cat) in a circular diagram^35^. (**b**) The circular diagram used to assess whether the participant’s arrow lies in the correct quadrant (i.e., 0°–90°, 90°–180°, 180°–270°, 270°–360°). (**c**) The black arrow shows the correct direction, the participant’s response is shown in blue. The red angle represents the participant’s angular error.

The Perspective-Taking/Spatial Orientation Test (PTSOT) is a paper-and-pencil test of perspective taking originally developed by Kozhevnikov and Hegarty^51^ and later modified by Hegarty and Waller^35^. During the test, the experimenter shows the participants an array of seven objects (e.g., a flower, a tree, a cat, etc.) in the upper half of a paper sheet. The participants are asked to imagine standing at one object (e.g., the flower) and facing another (e.g., the tree). The participants’ task is to indicate the direction of a third object (the target object, e.g., the cat). The lower half of the sheet displays a circle where the station point (the flower) is positioned in the center of the circle, and the imagined heading (the tree) is shown at the end of an arrow pointing upwards. Participants are asked to indicate the direction of the target object by drawing a line from the center of the circle in the direction believed to be correct. An example of the task is shown in Fig. 1a. There are 12 items in this test, one on each page, with varying angles of the target object direction. For each item, the array of objects is shown at the top of the page and the arrow circle is shown at the bottom. The experimenter instructs the participants not to physically rotate either the sheet or their own head or body. Participants have no time limit for completing the test.

Each item was scored in two ways. First, we assessed whether or not the participant’s arrow lies in the correct quadrant of the circle (i.e., 0°–90°, 90°–180°, 180°–270°, 270°–360°, shown in Fig. 1b). The percentage of correct responses from all 12 situations was then calculated. Second, the angular deviation in degrees between the participant’s arrow and the correct response was measured for each situation, and the mean angular error was calculated (shown in Fig. 1c).

It is important to note that several ataxia patients were unable to draw the arrow due to their motor disability. They were, however, able to indicate the direction on the circle with their finger, and the experimenter then drew the arrow for them.

### Directional-approach task

The Directional-approach Task is a component of the Navigation Test Suite, a well-established, virtual, realistic-looking battery of spatial navigation assessments^36,52^. The test takes place in a virtual environment that consists of streets with residential houses and four-way intersections. The houses bordering the streets are all identical, except for the unique houses (i.e., distinct landmarks) that are located at each intersection.

**Fig. 2:**
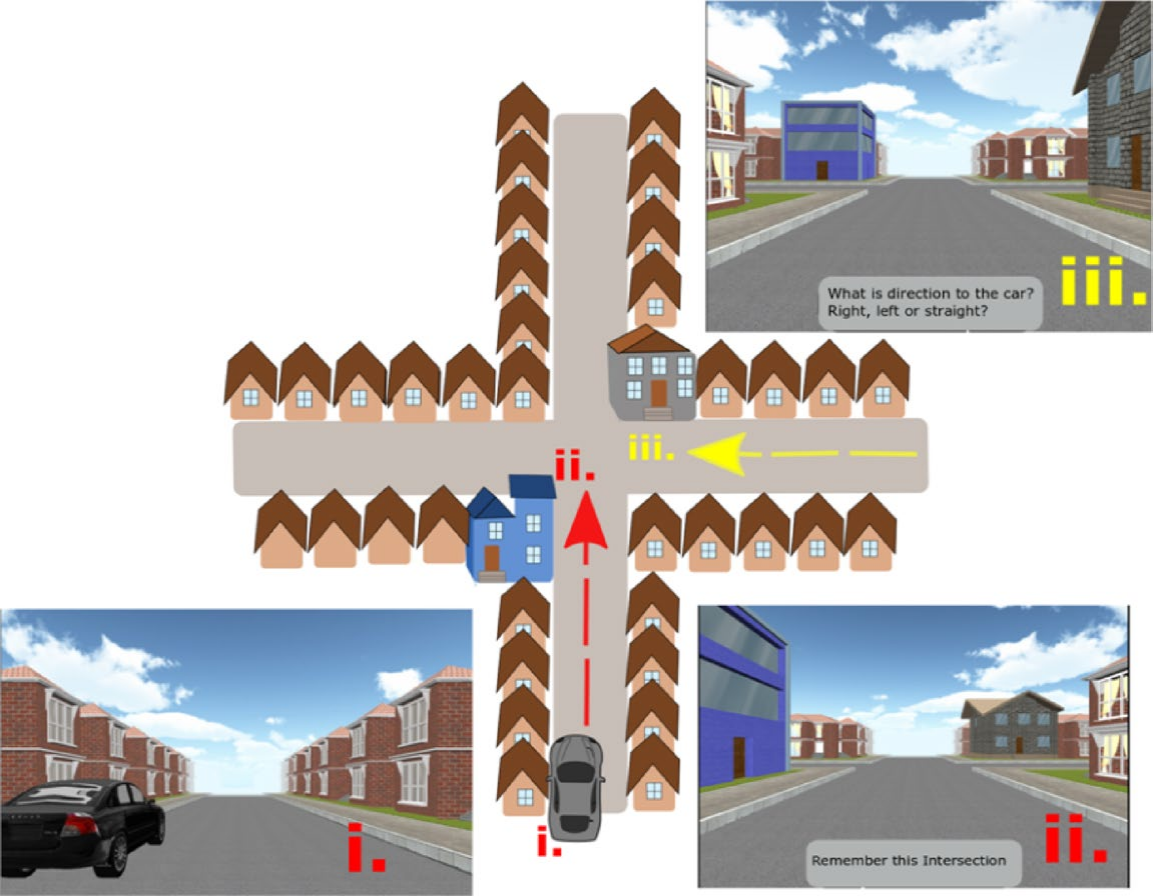
Schematic example and screenshots of the Directional-approach task—(i) The starting position next to the car, where participants begin the task. (ii) The encoding phase, where participants are passively transported toward one of the intersections featuring two unique houses. Participants have to remember where the car was parked. (iii) The test phase, where participants approach the intersection from a different direction (in this case from the east) and have to indicate the direction to the car^52^.

The Directional-approach task (Fig. 2) examines participants’ ability to encode the configuration of houses (landmarks) at an intersection, assessing perspective taking^53,54^. The task comprises 18 independent trials, each consisting of an encoding phase and a test phase:

1. Encoding Phase: Participants are placed in a virtual street next to a parked black car. They are passively transported toward an intersection featuring two unique houses (i.e., landmarks) at diagonally opposite corners. The remaining corners of the intersection have the same houses as those along the entire street. The movement stops 20 m before the center of the intersection, providing a clear view of the unique landmarks. Participants´ task is to memorize which street the car is parked on (south of the intersection in all trials).
2. Test Phase: Participants are passively transported toward the same intersection but approach it from a different street (west, east, or north). The movement again stops 20 m before the center of the intersection, so that both unique houses are visible. The participants are then asked to indicate the direction (left, right, or straight ahead) in which the car is parked (i.e., to indicate the street from which they originally approached the intersection). There is no time limit on completion.

The task requires participants to perform perspective shifts to align their current view with that of the encoding phase. The magnitude of the perspective shift depends on the approach direction during the test phase:

- 90° shift when approaching from the west or east
- 180° shift when approaching from the north

The participants were not aware of these cardinal directions, but the information was used in the analysis.

Participants were asked about their navigation strategy after the Directional-approach task. Reported strategies were classified into three groups: (1) Unique houses, remembering the position of one or two unique houses at the intersections, (2) more houses, remembering three, all four, or non-unique houses, and (3) non-specific, (e.g., guessing, remembering grass, which was the same at all intersections)^52^.

### Santa Barbara Sense of Direction Scale

We used the Czech version of the Santa Barbara Sense of Direction Scale (SBSOD) to assess self-reported spatial navigation abilities^30,37^. This questionnaire consists of 15 statements related to spatial navigation (e.g., “I am very good at giving directions”, “I very easily get lost in a new city”). Participants report their agreement with each statement by choosing a number on a Likert-type scale from 1 (“strongly agree”) to 7 (“strongly disagree”). When calculating the results, we reversed the scores on negatively worded statements so that the higher score always indicated better spatial navigation skills. The composite score (SBSOD composite) was calculated as the average score of all responses.

### Statistical analysis

Descriptive statistics were used to characterize the study sample using means and standard deviations (Table 1). One-way analyses of variance (ANOVAs) with post hoc false discovery rate (FDR) corrections (for 3 between-group comparisons—SCA vs. FRDA vs. HC) and chi-squared tests were used to examine between-group differences in demographic characteristics. T-tests were used to examine differences in FA-ADL and SARA scores between the SCA and FRDA groups. General linear models (GLMs) controlling for the effects of age, sex, and years of education were used to examine differences in each cognitive domain and SBSOD scores between the SCA and HC groups. Linear mixed models (LMMs) with random intercepts and trials as repeated measures, controlled for age, sex, and years of education, were used to examine differences between all groups in PTSOT angular deviations and correct quadrants (FDR corrected for 3 between-group comparisons) and controlled for age, sex, and years of education and BDI between the SCA and HC groups in Directional-approach Task scores. We then repeated these GLM and LMM analyses, controlling for MMSE to account for the between-group differences in global cognition. To examine the role of perspective shift in the Directional-approach Task, we repeated the LMM analysis and included perspective angle (90° vs. 180°) and the interaction between group status and perspective angle as independent variables in the model. Pearson point bivariate correlation and a chi-squared test were used to examine the associations between the employed strategy (unique houses vs. non-unique houses/non-specific) and Directional-approach Task scores and the between-group differences in employed strategies, respectively. LMMs with random intercepts and trials as repeated measures, controlled for age, sex, and years of education, were used to estimate the associations of PTSOT angular deviations and correct quadrants (outcome variables) with MMSE, SARA, and FA-ADL scores (independent variables) in the SCA and FRDA groups separately, and with SBSOD scores and each cognitive domain (independent variables) in the SCA group. These LMMs were also used to estimate the associations of Directional-approach Task scores (outcome variables) with MMSE, SARA, FA-ADL, and SBSOD scores and each cognitive domain (independent variables) in the SCA group. Multiple linear regressions controlled for age, sex, and years of education were used to estimate the associations of SBSOD scores (outcome variables) with MMSE, SARA, and FA-ADL scores and each cognitive domain (independent variables) in the SCA group. FDR correction was used in the association analyses to adjust for multiple comparisons, correcting for all independent variables associated with a given outcome variable. A two-tailed *p* < 0.05 was considered statistically significant. Analyses were performed using IBM SPSS Statistics 28.0.

**Table 1:** Demographic and spatial navigation data.

	SCA (n = 30)	FRDA (n = 30)	HC (n = 34)	*P*
Demographic data				
Age (years)	49.23 (15.41)**^++^	34.13 (13.65)	39.71 (10.96)	< .001
Women, n (%)	18 (60)	12 (40)*	24 (71)	.045
Education (years)	14.60 (3.19)	13.53 (3.26)**	15.74 (3.04)	.024
MMSE (score)	27.63 (2.22)***^+^	28.53 (1.74)	29.06 (0.81)	.004
BDI (score)	9.87 (7.04)	N/A	3.71 (4.53)	< .001
SARA (score)	10.60 (7.46)	21.83 (7.56)	N/A	< .001
FA-ADL (score)	6.67 (5.97)	15.70 (6.35)	N/A	< .001
Spatial navigation performance				
PTSOT angular deviations (degrees)^a^	64.50 (34.49)***^+++^	41.69 (29.21)	26.01 (15.42)	< .001
PTSOT correct quadrants (%)^a^	47.62 (29.65)**	67.22 (28.78)**	82.84 (16.02)	< .001
Directional-approach Task (score)^b^	10.43 (4.66)***	N/A	15.76 (2.09)	< .001
SBSOD (score)^a^	4.34 (1.02)	N/A	4.82 (1.00)	.054

Values are mean (SD) except for gender. Significant differences between the groups based on post-hoc analyses. *p < .05; **p < .01, and ***p < .001 compared to the HC group; ^+^*p* < .05; ^+++^*p* < .001 compared to the FRDA group.

SCA, Spinocerebellar ataxias; FRDA, Friedreich ataxia; HC, Healthy controls; MMSE, Mini-Mental State Examination; SARA, Scale for the Assessment and Rating of Ataxia; FA-ADL, Activities of Daily Living Scale; PTSOT, Perspective Taking/Spatial Orientation Test; SBSOD, Santa Barbara Sense of Direction Scale.

^a^Values are controlled for age, sex, and education.

^b^Values are controlled for age, sex, education, and BDI. *P* values refer to the main effect across the groups.

## Results

The characteristics of the participants are presented in Table 1. SCA participants were significantly older than both FRDA participants and HC (*p* ≤ 0.005). Additionally, SCA participants scored lower on the MMSE compared to FRDA participants and HC (*p* = 0.039 and *p* < 0.001, respectively). FRDA participants were less educated than HC (*p* = 0.007). The proportion of females was smaller among FRDA participants compared to HC (*p* = 0.045). SCA participants exhibited less severe impairment on SARA and FA-ADL scores compared to FRDA participants (*p* < 0.001 for both). SCA participants performed worse than HC across all cognitive domains (*p* < 0.001)*.*

### Perspective-taking/spatial orientation test (PTSOT)

**Fig. 3:**
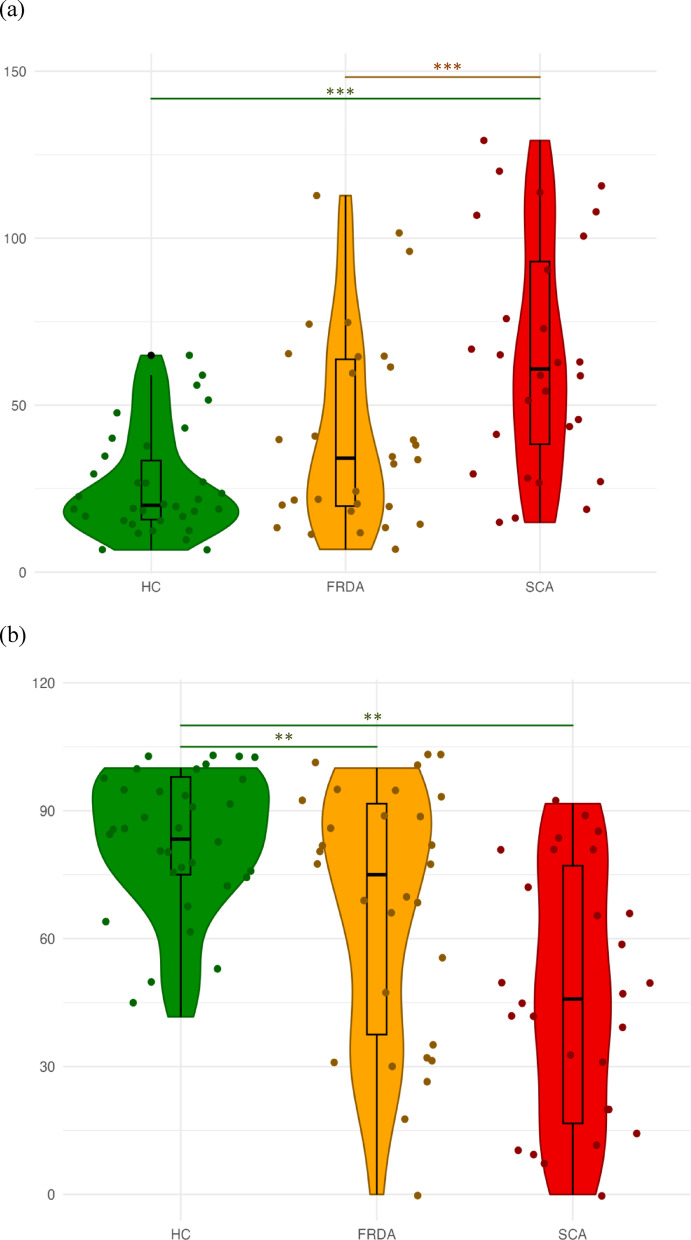
(**a**) PTSOT—Mean angular deviations in degrees. (**b**) PTSOT—Mean percentage of responses in the correct quadrant of the circular diagram. Group comparisons are adjusted for age, sex, and education. SCA, Spinocerebellar ataxias; FRDA, Friedreich ataxia; HC, Healthy controls, *p < .05; **p < .01; ***p < .001.

On the PTSOT**,** SCA participants showed greater angular deviations compared to both HC and FRDA participants (*p* < 0.001 for both), while FRDA participants performed similarly to HC. These differences remained significant after controlling for global cognition (data in the Supplementary Table 2). SCA participants identified fewer correct quadrants than HC (*p* = 0.007) and FRDA participants (*p* = 0.054). The latter result approached statistical significance. FRDA participants also identified fewer correct quadrants than HC (*p* = 0.007). The difference between SCA and FRDA participants was not significant after adjusting for global cognition. PTSOT results are shown in Fig. 3.

### Directional-approach task

**Fig. 4:**
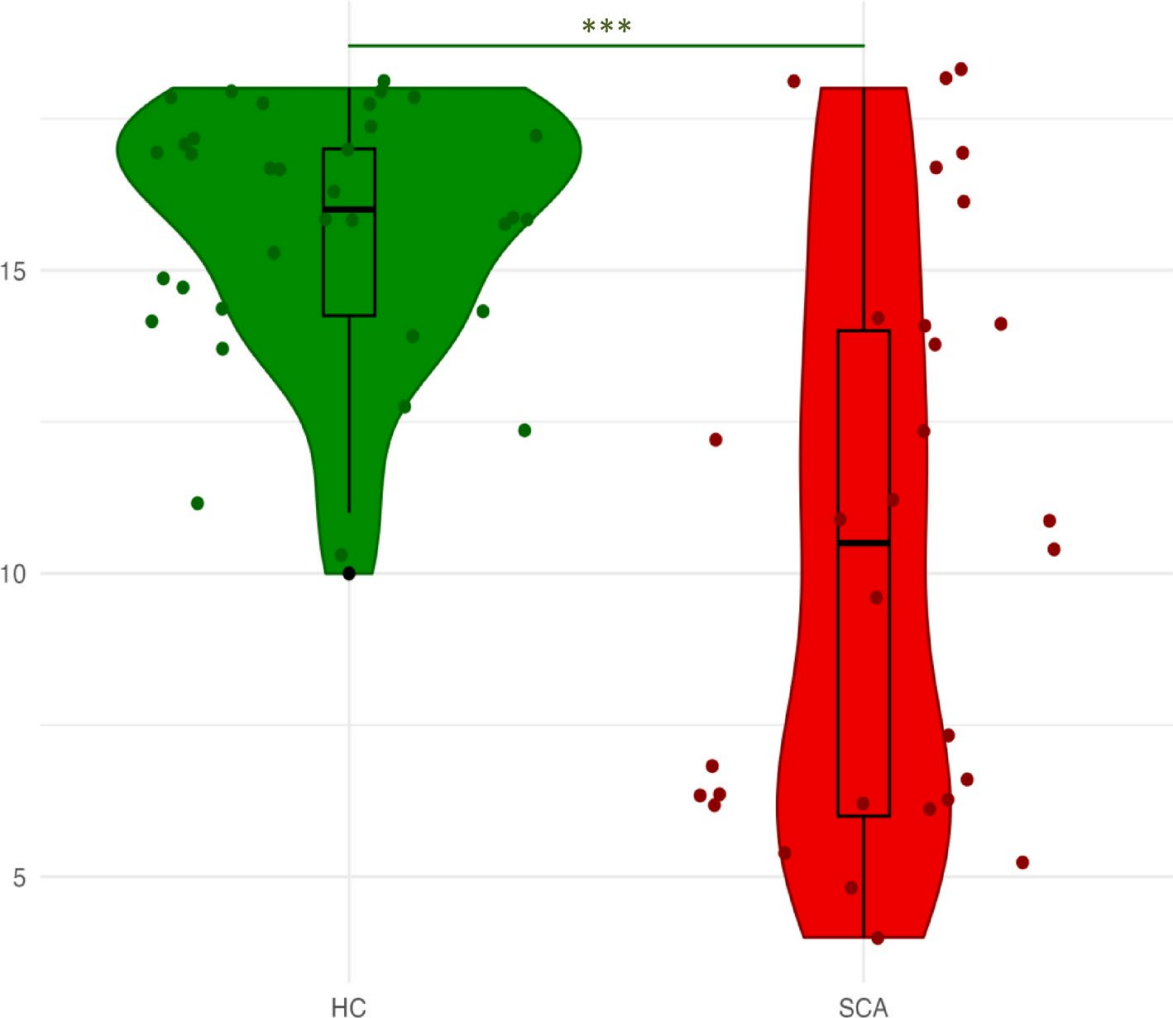
Directional-approach task—mean number of correct responses. Group comparisons are adjusted for age, sex, education, and BDI. SCA, Spinocerebellar ataxias; HC, Healthy controls, *p < .05; **p < .01; ***p < .001.

In the Directional-approach Task (Fig. 4)**,** SCA participants performed worse than HC (*p* < 0.001), with results remaining significant after controlling for global cognition (data in the Supplementary Table 2). Performance across all participants was worse during trials requiring a 180° perspective shift compared to a 90° shift (*p* = 0.008). However, the interaction between group status and perspective angle was not significant (*p* = 0.135). The SCA participants used a specific strategy with unique houses less frequently than HC (70% vs. 94%, *p* = 0.011), and not using this strategy was associated with worse task performance (*r*_*pb*_ = 0.38, *p* = 0.002).

### Santa Barbara Sense of Direction Scale (SBSOD)

In the SBSOD, SCA participants reported worse spatial navigation abilities than HC. This result approached statistical significance (*p* = 0.054). The difference was not significant after adjusting for global cognition.

### Associations between cognition, motor impairment, and perspective taking

In both SCA and FRDA participants, global cognition was significantly associated with perspective taking performance on the PTSOT, as reflected in angular deviations (β =  − 7.93, *p* = 0.009; β =  − 6.95, *p* = 0.006) and correct quadrants (β = 0.07, *p* = 0.009; β = 0.09, *p* = 0.001). However, no significant associations were observed between SARA or FA-ADL scores and PTSOT performance in either group.

The results from the neuropsychological tests are presented in Table 2, and composite Z-scores for all cognitive domains are presented in Supplementary Table 1. In SCA participants, perspective taking performance on the PTSOT was significantly associated with visuospatial functions (β =  − 13.89, *p* = 0.040; β = 0.12, *p* = 0.038), executive functions (β =  − 24.87, *p* < 0.001; β = 0.20, *p* = 0.003), and attention and working memory (β =  − 16.07, *p* = 0.038; β = 0.14, *p* = 0.040), but not with verbal memory or language. After correction for multiple comparisons, associations with visuospatial functions and attention and working memory were no longer significant.

**Table 2:** Neuropsychological data.

	SCA (n = 30)	HC (n = 34)	*P*
AVLT 1–5 (score)	46.48 (10.28, 21–70)	57.97 (7.28, 40–70)	< .001
AVLT 30 (score)	9.46 (3.23, 2–15)	12.41 (1.97, 7–15)	< .001
LM—delayed recall (score)	14.39 (4.16, 5–25)	19.06 (2.74, 12–24)	< .001
JLO (score)	24.15 (3.33, 19–30)	27.26 (2.09, 22–30)	< .001
TMT B (s)	201.48 (239.16, 52–999)	58.29 (15.34, 33–91)	< .001
WCST (perseverative errors)	12.75 (7.57, 3–38)	6.32 (2.92, 4–15)	< .001
COWAT (score)	37.11 (10.33, 17–62)	53.79 (11.99, 31–80)	< .001
SCWT (number of correct items)	35.04 (12.24, 11–61)	49.74 (13.18, 29–84)	< .001
TMT A (s)	58.63 (31.87, 19–145)	28.56 (8.71, 15–50)	< .001
F-Digit Span (score)	8.24 (1.83, 5–13)	9.29 (2.07, 6–15)	.038
R-Digit Span (score)	6.11 (2.41, 3–13)	7.41 (1.89, 4–13)	.020
BNT (score)	27.29 (2.40, 22–30)	29.26 (0.90, 27–30)	< .001
VF Animals (score)	19.82 (5.06, 9–31)	29.62 (5.16, 20–41)	< .001
VF Vegetable (score)	11.93 (3.44, 6–21)	16.44 (3.86, 10–25)	< .001

Values are mean (SD, range). All values are controlled for age, sex, and education. *P* values refer to the main effect across the groups.

SCA, Spinocerebellar ataxias; FRDA, Friedreich ataxia; HC, Healthy controls; AVLT, Rey Auditory Verbal Learning Test; AVLT 1–5, Trials 1 to 5 total; AVLT 30, Delayed word recall after 30 min; LM-delayed recall, Logical Memory delayed recall; JLO, Judgement of Line Orientation; TMT A and B, Trail Making Tests A and B; WCST, Wisconsin Card Sorting Test; COWAT, Controlled Oral Word Association Test (Czech version with letters K, P, and S); SCWT, Stroop Color and Word Test; F-Digit Span, WAIS-III Digit Span Forward; R-Digit Span WAIS-III Digit Span Reversed; BNT, Boston Naming Test; VF, Verbal fluency.

For the Directional-approach Task, global cognition was significantly associated with performance in SCA participants (β = 0.05, *p* = 0.013), whereas no associations were found with SARA or FA-ADL scores. Directional-approach Task performance in SCA participants was also associated with visuospatial functions (β = 0.16, *p* < 0.001), executive functions (β = 0.21, *p* < 0.001), and attention and working memory (β = 0.18, *p* = 0.001), but not with verbal memory or language. These associations remained significant after correction for multiple comparisons.

In SCA participants, self-reported spatial navigation abilities, as measured by the SBSOD, showed no significant associations with MMSE, SARA, FA-ADL scores, PTSOT angular deviations and correct quadrants identified, Directional-approach Task scores, or cognitive domains except for language (β = 0.58, *p* = 0.030). After correction for multiple comparisons, the association with language was no longer significant.

## Discussion

In this study, we focused on spatial perspective taking, an important aspect of spatial navigation, in patients with degenerative ataxias and its relation to cognitive functions, motor impairment, and self-reported spatial navigation abilities.

We found that SCA patients performed significantly worse than HC on both perspective taking tests. In contrast, FRDA patients demonstrated only partial deficits relative to HC. The pronounced disruption in spatial perspective taking in SCA was associated with global cognitive performance and several specific cognitive domains, but not with cerebellar motor impairment or FA-ADL.

These findings support the growing evidence that the cerebellum plays a crucial role in navigation, including spatial perspective taking. Our study is the first to demonstrate deficits in this ability in SCA patients. These results align with research in animal models of cerebellar damage, which have shown impaired spatial navigation in mice with cerebellar lesions, including the mouse model of SCA1^55–57^. Our results are also in line with previous findings in several patients with cerebellar tumors or stroke, who exhibited impairment in mental rotation, an ability distinct from perspective taking, yet closely related to it^27,35^. It should be noted that worse performance on the more challenging trials with the 180° perspective shift than with the 90° shift indicates that the impairment in the Directional-approach Task is indeed determined by impaired perspective taking ability rather than other factors (e.g., severity of cognitive impairment, lack of attention, etc.). Interestingly, SCA patients were more likely to rely on non-specific or suboptimal strategies during the Directional-approach task compared to healthy controls, and the use of such strategies was significantly associated with poorer task performance. This pattern may reflect executive dysfunction, which is supported by the observed associations between task performance and executive functions scores in our cohort.

The observed impairment in perspective taking may not exclusively reflect a deficit in spatial transformation per se, but could be part of a broader disruption in cerebellar-supported cognitive functions. The cerebellum is increasingly recognized as a structure involved in building internal forward models that enable the prediction of sensory consequences of actions and the coordination of temporally structured sequences, both in the motor and cognitive domains. These internal models are thought to support mental simulation and flexible manipulation of internal representations, the functions potentially relevant for imagining spatial perspectives and anticipating spatial configurations from different orientations^58–60^.

Perspective taking is a crucial component of spatial navigation, a fundamental skill in daily life. Impairments in spatial navigation are associated with increased dependency, reduced autonomy, and a heightened risk of getting lost, all of which can significantly diminish the quality of life^29,61^. The mean score of 10.43 out of 18 on the Directional-Approach task in our SCA patients is only marginally better than chance, indicating a substantial impairment.

The severity of the perspective taking impairment in SCA patients in our results, as measured by the Directional-approach Task (mean percentage of correct answers 58%), was similar in magnitude to that of patients with amnestic mild cognitive impairment (aMCI) due to a non-Alzheimer pathology (54% correct) and was only slightly milder than that of aMCI patients due to Alzheimer disease (44.6% correct) in a study using a shorter version of the same test^62^. This comparison with a neurodegenerative disorder known for its marked impairment of spatial navigation puts our results in context and suggests a possible clinical significance of our findings in ataxia patients.

In contrast to SCA patients, the FRDA group showed less severe impairment despite the more pronounced motor disability. This is analogous to the less severe cognitive, neuropsychiatric, and also social cognitive impairment in FRDA patients compared to SCA found in previous studies^6,63^. The difference may stem from the less severe cerebellar grey matter atrophy in FRDA^64^. However, the potential involvement of extra-cerebellar structures, such as the basal ganglia, on these non-motor manifestations in SCA cannot be ruled out^65^.

In accordance with our hypotheses, we found that the perspective taking impairment in SCA patients was associated with general cognition and specific cognitive domains, namely visuospatial functions, executive functions, and attention and working memory. These domains are related to spatial navigation and form the core of the cerebellar cognitive affective syndrome^2,26^.

In addition, the perspective taking ability in SCA patients did not correlate with cerebellar motor impairment as measured by the SARA or with motor functional impairment as measured by the FA-ADL scale. This dissociation reflects previous findings in cerebellar cognitive affective syndrome, such as neuropsychiatric symptoms and deficits in social cognition, which likewise show no direct relationship with motor impairment^10,66^. While this dissociation is not always consistently observed in neuropsychological tests, the overall evidence confirms that distinct cerebellar regions regulate motor and cognitive functions^9,67–69^. Neuroimaging studies further support this distinction, identifying separate motor and cognitive-affective areas within the cerebellum^3,70^. Notably, functional neuroimaging studies in healthy individuals have shown that spatial navigation, including perspective taking, primarily activates the posterior cerebellar lobe—a region associated with cognitive processing^14^. Our results from the perspective taking tests fit well into this picture.

Interestingly, despite the objectively identified deficits in spatial perspective taking, SCA patients reported only slightly worse spatial navigation abilities compared to HC on the self-reported questionnaire. Their self-reported navigation abilities were not associated with performance on the Directional-approach Task or with performance on the PTSOT, as measured by angular deviations and correct quadrants identified. Previous studies in healthy individuals have shown that self-reported navigation ability is linked to spatial task performance^71–73^, including perspective taking tests, particularly the PTSOT^51,74^. However, in clinical population, self-reported, unlike informant-reported measures fail to reliably distinguish cognitively impaired individuals from HC, possibly due to reduced awareness of cognitive deficits or difficulties in accurately assessing one’s own abilities^75,76^. In SCA patients, this partial lack of awareness may be further influenced by “dysmetria of thought” a phenomenon observed in cerebellar disorders^2^. Moreover, given that motor impairment in ataxia is the primary perceived and functionally limiting difficulty in patients with ataxia, it may dominate the patients’ perception of their overall disability. Consequently, cognitive difficulties—such as impairments in spatial perspective taking—may be perceived as less relevant or may go unnoticed. Another possible contributing factor is that patients with significant motor impairment tend to travel less frequently, and while travelling, they are often accompanied and even physically supported by their caregivers, who may take on the responsibility for spatial navigation and the patients may thus have fewer opportunities to evaluate their navigation skills in real-world settings. As a result, self-reported spatial navigation questionnaires may not be reliable indicators of cognitive performance in individuals with cerebellar disorders and restricted mobility.

The main strength of this study is its novelty, as it is the first study to investigate spatial perspective taking, a key component of spatial navigation, in patients with degenerative ataxias, or indeed any cerebellar disorder. By employing well-established and ecologically valid tests, alongside a comprehensive neuropsychological assessment, the study provides valuable insights into the cognitive deficits associated with ataxias. Additionally, the comparison of SCA and FRDA patients highlights distinct patterns of cognitive and perspective taking impairments in these conditions.

However, several limitations must be acknowledged. The relatively small sample size, a challenge inherent to studies on rare diseases, limits the generalizability of our findings. The heterogeneity within the SCA group, which included various genetic subtypes, further complicates the interpretation and limits the possibility to assess differences between various SCA subtypes. Moreover, the FRDA group underwent a less comprehensive testing protocol, which may have influenced the comparability of results. Finally, the absence of neuroimaging data prevents direct correlations between cognitive deficits and cerebellar or extra-cerebellar structural changes. Future studies should aim to address these limitations by including larger, more genetically homogenous cohorts, taking also age of onset and disease duration into account, and, importantly, incorporating advanced neuroimaging techniques such as voxel-based morphometry or functional connectivity studies to explore the structural–functional correlates of perspective taking abilities. Additionally, future studies might explore the discrepancy between spatial navigation deficits and patients’ self-awareness of these deficits in greater detail, employing informant-rated measures as well. Studies aimed at evaluating spatial navigation across a broader spectrum, with a focus on aspects beyond perspective taking, are currently underway.

## Conclusion

This study is the first to demonstrate impairments in spatial perspective taking, an important subcomponent of spatial navigation, in SCA patients with deficits of similar magnitude to those observed in dementing conditions. Impaired perspective taking was associated with cognitive deficits but not with motor impairment. The milder deficits observed in FRDA patients align with the less pronounced cerebellar atrophy compared to SCA. Our findings expand the understanding of the cognitive impairments in SCA, highlighting the functional dissociation between motor and non-motor cerebellum. Furthermore, our findings support the presence of clinically significant spatial navigation deficits in SCA, adding to the existing knowledge of complex cognitive dysfunction in these disorders.

## Supplementary Information

Below is the link to the electronic supplementary material.

Supplementary Material 1

## Data Availability

Data are available from the corresponding author upon reasonable request.

## References

[1] Jayadev, S. & Bird, T. D. Hereditary ataxias: Overview. *Genet. Med.* **15**(9), 673–683 (2013). 23538602 10.1038/gim.2013.28

[2] Argyropoulos, G. P. D. et al. The cerebellar cognitive affective/schmahmann syndrome: A task force paper. *Cerebellum* **19**(1), 102–125 (2020). 31522332 10.1007/s12311-019-01068-8 PMC6978293

[3] O’Reilly, J. X. et al. Distinct and overlapping functional zones in the cerebellum defined by resting state functional connectivity. *Cereb. Cortex* **20**(4), 953–965 (2010). 19684249 10.1093/cercor/bhp157 PMC2837094

[4] Krienen, F. M. & Buckner, R. L. Segregated fronto-cerebellar circuits revealed by intrinsic functional connectivity. *Cereb. Cortex* **19**(10), 2485–2497 (2009). 19592571 10.1093/cercor/bhp135 PMC2742600

[5] Bürk, K. Cognition in hereditary ataxia. *Cerebellum* **6**(3), 280–286 (2007). 17786824 10.1080/14734220601115924

[6] Sayah, S. et al. Personality and neuropsychological profiles in Friedreich ataxia. *Cerebellum* **17**(2), 204–212 (2018). 29086357 10.1007/s12311-017-0890-5

[7] Schmahmann, J. The cerebellar cognitive affective syndrome. *Brain* **121**(4), 561–579. 10.1093/brain/121.4.561 (1998). 9577385 10.1093/brain/121.4.561

[8] Pira, F. et al. Dissociation between motor and cognitive impairments in SCA2: Evidence from a follow-up study. *J. Neurol.* **254**(10), 1455–1456 (2007). 17680296 10.1007/s00415-007-0548-1

[9] Fancellu, R. et al. Longitudinal study of cognitive and psychiatric functions in spinocerebellar ataxia types 1 and 2. *J. Neurol.* **260**(12), 3134–3143 (2013). 24122064 10.1007/s00415-013-7138-1

[10] Karamazovova, S., Matuskova, V., Ismail, Z. & Vyhnalek, M. Neuropsychiatric symptoms in spinocerebellar ataxias and Friedreich ataxia. *Neurosci. Biobehav. Rev.* **150**, 105205 (2023). 37137435 10.1016/j.neubiorev.2023.105205

[11] Rodríguez-Labrada, R. et al. Cognitive decline is closely associated with ataxia severity in spinocerebellar ataxia type 2: A validation study of the Schmahmann syndrome scale. *Cerebellum* **21**(3), 391–403 (2022). 34313938 10.1007/s12311-021-01305-z

[12] Maas, R. P. P. W. M., Killaars, S., van de Warrenburg, B. P. C. & Schutter, D. J. L. G. The cerebellar cognitive affective syndrome scale reveals early neuropsychological deficits in SCA3 patients. *J. Neurol.* **268**(9), 3456–3466 (2021). 33743045 10.1007/s00415-021-10516-7 PMC8357713

[13] Lefort, J. M., Rochefort, C. & Rondi-Reig, L. Cerebellar contribution to spatial navigation: New insights into potential mechanisms. *Cerebellum* **14**(1), 59–62 (2015). 25630873 10.1007/s12311-015-0653-0

[14] Iglói, K. et al. Interaction between hippocampus and cerebellum crus I in sequence-based but not place-based navigation. *Cereb. Cortex* **25**(11), 4146–4154 (2015). 24947462 10.1093/cercor/bhu132 PMC4886832

[15] Rochefort, C., Lefort, J. & Rondi-Reig, L. The cerebellum: A new key structure in the navigation system. *Front. Neural Circuits*10.3389/fncir.2013.00035/abstract (2013). 23493515 10.3389/fncir.2013.00035 PMC3595517

[16] Zacks, J. M. & Michelon, P. Transformations of visuospatial images. *Behav. Cogn. Neurosci. Rev.* **4**(2), 96–118 (2005). 16251727 10.1177/1534582305281085

[17] Lambrey, S. et al. Distinct visual perspective-taking strategies involve the left and right medial temporal lobe structures differently. *Brain* **131**(2), 523–534 (2008). 18178570 10.1093/brain/awm317

[18] Marková, H. et al. Perspective taking abilities in amnestic mild cognitive impairment and Alzheimer’s disease. *Behav. Brain Res.* **281**, 229–238 (2015). 25541035 10.1016/j.bbr.2014.12.033

[19] Laczó, M. et al. Different profiles of spatial navigation deficits in Alzheimer’s disease biomarker-positive versus biomarker-negative older adults with amnestic mild cognitive impairment. *Front. Aging Neurosci.* **14**, 886778 (2022). 35721017 10.3389/fnagi.2022.886778 PMC9201637

[20] Majerová, V. et al. Disturbance of real space navigation in moderately advanced but not in early Huntington’s disease. *J. Neurol. Sci.* **312**(1–2), 86–91 (2012). 21875725 10.1016/j.jns.2011.08.016

[21] Glikmann-Johnston, Y. et al. Hippocampal and striatal volumes correlate with spatial memory impairment in Huntington’s disease. *J. Neurosci. Res.* **99**(11), 2948–2963 (2021). 34516012 10.1002/jnr.24966

[22] Fernandez-Baizan, C. et al. Patients with Parkinson’s disease show alteration in their visuospatial abilities and in their egocentric and allocentric spatial orientation measured by card placing tests. *J. Parkinsons Dis.* **10**(4), 1807–1816 (2020). 33016894 10.3233/JPD-202122

[23] Schneider, C. B. et al. Spatial learning deficits in Parkinson’s disease with and without mild cognitive impairment. *Parkinsonism Relat. Disord.* **36**, 83–88 (2017). 28027851 10.1016/j.parkreldis.2016.12.020

[24] Kaiser, S. et al. Gender-specific strategy use and neural correlates in a spatial perspective taking task. *Neuropsychologia* **46**(10), 2524–2531 (2008). 18514745 10.1016/j.neuropsychologia.2008.04.013

[25] Creem-Regehr, S. H., Neil, J. A. & Yeh, H. J. Neural correlates of two imagined egocentric transformations. *Neuroimage* **35**(2), 916–927 (2007). 17275336 10.1016/j.neuroimage.2006.11.057

[26] Wolbers, T. & Hegarty, M. What determines our navigational abilities?. *Trends Cogn. Sci.* **14**(3), 138–146 (2010). 20138795 10.1016/j.tics.2010.01.001

[27] Wallesch, C.-W. & Horn, A. Long-term effects of cerebellar pathology on cognitive functions. *Brain Cogn.* **14**(1), 19–25 (1990). 2223042 10.1016/0278-2626(90)90057-u

[28] Molinari, M., Petrosini, L., Misciagna, S. & Leggio, M. G. Visuospatial abilities in cerebellar disorders. *J Neurol Neurosurg Psychiatry* **75**(2), 235–240 (2004). 14742596 PMC1738892

[29] Ham, I., Kant, N., Postma, A. & Visser-Meily, J. Is navigation ability a problem in mild stroke patients? Insights from self-reported navigation measures. *J. Rehabil. Med.* **45**(5), 429–433 (2013). 23615778 10.2340/16501977-1139

[30] Laczó, M. et al. Spatial navigation questionnaires as a supportive diagnostic tool in early Alzheimer’s disease. *iScience* **27**(6), 109832 (2024). 38779476 10.1016/j.isci.2024.109832 PMC11108981

[31] Allison, S. et al. Alzheimer disease biomarkers and driving in clinically normal older adults. *Alzheimer Dis. Assoc. Disord.* **32**(2), 101–106 (2018). 29578861 10.1097/WAD.0000000000000257 PMC5963990

[32] Allison, S. L. et al. Developing a spatial navigation screening tool sensitive to the preclinical Alzheimer disease continuum. *Arch. Clin. Neuropsychol.* **34**(7), 1138–1155 (2019). 31197326 10.1093/arclin/acz019 PMC6849466

[33] Schmitz-Hübsch, T. et al. Scale for the assessment and rating of ataxia. *Neurology* **66**(11), 1717–1720 (2006). 16769946 10.1212/01.wnl.0000219042.60538.92

[34] Bürk, K., Schulz, S. R. & Schulz, J. B. Monitoring progression in Friedreich ataxia: The use of clinical scales. *J. Neurochem.* **126**(s1), 118–124 (2013). 23859347 10.1111/jnc.12318

[35] Hegarty, M. A dissociation between mental rotation and perspective-taking spatial abilities. *Intelligence* **32**(2), 175–191 (2004).

[36] Wiener, J. M. et al. A novel virtual-reality-based route-learning test suite: Assessing the effects of cognitive aging on navigation. *Behav. Res. Methods* **52**(2), 630–640 (2020). 31236900 10.3758/s13428-019-01264-8 PMC7148270

[37] Hegarty, M. Development of a self-report measure of environmental spatial ability. *Intelligence* **30**(5), 425–447 (2002).

[38] Nasreddine, Z. S. et al. The Montreal cognitive assessment, MoCA: A brief screening tool for mild cognitive impairment. *J. Am. Geriatr. Soc.* **53**(4), 695–699 (2005). 15817019 10.1111/j.1532-5415.2005.53221.x

[39] Fasnacht, J. S. et al. Conversion between the Montreal cognitive assessment and the mini-mental status examination. *J. Am. Geriatr. Soc.* **71**(3), 869–879. 10.1111/jgs.18124 (2023). 36346002 10.1111/jgs.18124

[40] Štěpánková, H. et al. Mini-mental state examination—Czech normative study. *Česká a slovenská neurologie a neurochirurgie* **78**(111), 57–63 (2015).

[41] Bezdicek, O. et al. Czech version of Rey Auditory Verbal Learning test: Normative data. *Aging Neuropsychol. Cogn.* **21**(6), 693–721 (2014). 10.1080/13825585.2013.865699 24344673

[42] Nikolai, T. et al. The uniform data set, Czech version: Normative data in older adults from an international perspective. *J. Alzheimer’s Dis.* **61**(3), 1233–1240 (2018). 29332045 10.3233/JAD-170595 PMC6939612

[43] Nikolai, T. et al. Tests of verbal fluency, Czech normative study in older patients. *Česká a Slovenská Neurologie a Neurochirurgie* **78/111**(3), 292–299 (2015).

[44] Kongs, K. S., Thompson, L. L., Iverson, G. L. & Heaton, R. K. Wisconsin card sorting test-64 card version (WCST-64). *Psychol. Assess. Resour.* (2000).

[45] Bezdicek, O. et al. Czech version of the trail making test: Normative data and clinical utility. *Arch. Clin. Neuropsychol.* **27**(8), 906–914 (2012). 23027441 10.1093/arclin/acs084

[46] Krivá, Ľ. Stroopův test. Hogrefe—Testcentrum (2013).

[47] Černochová, D. et al. *WAIS-III—Wechslerova Inteligenční Škála Pro Dospělé* (Hogrefe-Testcentrum, 2010).

[48] Smith, A. *Symbol Digit Modalities Test (SDMT). Manual (Revised)* (Western Psychological Services, Los Angeles, 1982).

[49] Benton, A., Hamsher, K. D., Varney, N. & Spreen, O. *Contribution to Neuropsychological Assessment* (Oxford University Press, 1983).

[50] Beck, A. T. et al. *Beck Depression Inventory* (PsycTESTS Dataset, 2011).

[51] Kozhevnikov, M. & Hegarty, M. A dissociation between object manipulation spatial ability and spatial orientation ability. *Mem. Cogn.* **29**(5), 745–756 (2001). 10.3758/bf03200477 11531229

[52] Laczó, M. et al. Spatial navigation and visuospatial strategies in typical and atypical aging. *Brain Sci.* **11**(11), 1421 (2021). 34827423 10.3390/brainsci11111421 PMC8615446

[53] Wiener, J. M., de Condappa, O., Harris, M. A. & Wolbers, T. Maladaptive bias for extrahippocampal navigation strategies in aging humans. *J. Neurosci.* **33**(14), 6012–6017 (2013). 23554482 10.1523/JNEUROSCI.0717-12.2013 PMC6618910

[54] de Condappa, O. & Wiener, J. M. Human place and response learning: Navigation strategy selection, pupil size and gaze behavior. *Psychol. Res.* **80**(1), 82–93 (2016). 25537525 10.1007/s00426-014-0642-9

[55] Colombel, C., Lalonde, R. & Caston, J. The effects of unilateral removal of the cerebellar hemispheres on spatial learning and memory in rats. *Brain Res.* **1004**(1–2), 108–115 (2004). 15033425 10.1016/j.brainres.2003.10.075

[56] Matilla, A. et al. Mice lacking ataxin-1 display learning deficits and decreased hippocampal paired-pulse facilitation. *J. Neurosci.* **18**(14), 5508–5516 (1998). 9651231 10.1523/JNEUROSCI.18-14-05508.1998 PMC6793485

[57] Gandhi, C. C., Kelly, R. M., Wiley, R. G. & Walsh, T. J. Impaired acquisition of a Morris water maze task following selective destruction of cerebellar purkinje cells with OX7-saporin. *Behav. Brain Res.* **109**(1), 37–47 (2000). 10699656 10.1016/s0166-4328(99)00160-6

[58] Ito, M. Control of mental activities by internal models in the cerebellum. *Nat. Rev. Neurosci.* **9**(4), 304–313 (2008). 18319727 10.1038/nrn2332

[59] Sokolov, A. A., Miall, R. C. & Ivry, R. B. The cerebellum: Adaptive prediction for movement and cognition. *Trends Cogn. Sci.* **21**(5), 313–332 (2017). 28385461 10.1016/j.tics.2017.02.005 PMC5477675

[60] Moberget, T. & Ivry, R. B. Cerebellar contributions to motor control and language comprehension: Searching for common computational principles. *Ann. N. Y. Acad. Sci.* **1369**(1), 154–171 (2016). 27206249 10.1111/nyas.13094 PMC5260470

[61] van der Ham, I. J. M., Koutzmpi, V., van der Kuil, M. N. A. & van der Hiele, K. Spatial navigation performance in people with multiple sclerosis-a large-scale online study. *Mult. Scler. Relat. Disord.* **58**, 103423 (2022). 35216792 10.1016/j.msard.2021.103423

[62] Laczó, M. et al. Different profiles of spatial navigation deficits in Alzheimer’s disease biomarker-positive versus biomarker-negative older adults with amnestic mild cognitive impairment. *Front. Aging Neurosci.* **14**, 886778 (2022). 35721017 10.3389/fnagi.2022.886778 PMC9201637

[63] Costabile, T. et al. Emotion recognition and psychological comorbidity in Friedreich’s ataxia. *Cerebellum* **17**(3), 336–345 (2018). 29327279 10.1007/s12311-018-0918-5

[64] Stefanescu, M. R. et al. Structural and functional MRI abnormalities of cerebellar cortex and nuclei in SCA3, SCA6 and Friedreich’s ataxia. *Brain* **138**(5), 1182–1197 (2015). 25818870 10.1093/brain/awv064 PMC5963415

[65] Reetz, K. et al. Genotype-specific patterns of atrophy progression are more sensitive than clinical decline in SCA1, SCA3 and SCA6. *Brain* **136**(3), 905–917 (2013). 23423669 10.1093/brain/aws369

[66] Karamazovova, S., Matuskova, V., Svecova, N. & Vyhnalek, M. Social cognition in degenerative cerebellar ataxias. *Curr. Opin. Behav. Sci.* **54**, 101313 (2023).

[67] Hernández-Torres, A. et al. Longitudinal study of cognitive functioning in Friedreich’s ataxia. *J. Int. Neuropsychol. Soc.* **27**(4), 343–350 (2021). 33050966 10.1017/S1355617720000958

[68] Gigante, A. F. et al. The relationships between ataxia and cognition in spinocerebellar ataxia type 2. *Cerebellum* **19**(1), 40–47 (2020). 31637587 10.1007/s12311-019-01079-5

[69] Klinke, I. et al. Neuropsychological features of patients with spinocerebellar ataxia (SCA) types 1, 2, 3, and 6. *Cerebellum* **9**(3), 433–442 (2010). 20502998 10.1007/s12311-010-0183-8 PMC2949561

[70] Buckner, R. L. et al. The organization of the human cerebellum estimated by intrinsic functional connectivity. *J. Neurophysiol.* **106**(5), 2322–2345 (2011). 21795627 10.1152/jn.00339.2011 PMC3214121

[71] Prestopnik, J. & Roskos-Ewoldsen, B. The relations among wayfinding strategy use, sense of direction, sex, familiarity, and wayfinding ability. *J. Environ. Psychol.* **20**(2), 177–191 (2000).

[72] Sholl, J., Acacio, J., Makar, R. & Christlan, L. The relation of sex and sense of direction to spatial orientation in an unfamiliar environment. *J. Environ. Psychol.* **20**(1), 17–28 (2000).

[73] Montello, D. R. & Pick, H. L. Jr. Integrating knowledge of vertically aligned large-scale spaces. *Environ. Behav.* **25**(3), 457–484 (1993).

[74] Weisberg, S. M. et al. Variations in cognitive maps: Understanding individual differences in navigation. *J. Exp. Psychol. Learn. Mem. Cogn.* **40**(3), 669–682 (2014). 24364725 10.1037/a0035261

[75] Nosheny, R. L. et al. The role of dyadic cognitive report and subjective cognitive decline in early ADRD clinical research and trials: Current knowledge, gaps, and recommendations. *Alzheimer’s Dement. Transl. Res. Clin. Interv.* **8**(1), e12357 (2022). 10.1002/trc2.12357 PMC9530696 36226046

[76] Roberts, J. L., Clare, L. & Woods, R. T. Subjective memory complaints and awareness of memory functioning in mild cognitive impairment: A systematic review. *Dement. Geriatr. Cogn. Disord.* **28**(2), 95–109 (2009). 19684399 10.1159/000234911

