# Supplementary material for: Spatial perspective taking is impaired in spinocerebellar ataxias and Friedreich ataxia
Source: Sci Rep. 2025 Aug 24;15:31126. doi: 10.1038/s41598-025-16302-z (PMC12375724; doi:10.1038/s41598-025-16302-z)
Supplement: Supplementary file 1 — Supplementary Material 1 [file 41598_2025_16302_MOESM1_ESM.docx]

**Supplementary Table 1: Composite Z-scores for all cognitive domains**

|  | SCA  (n = 30) | HC  (n = 34) | *P* |
| --- | --- | --- | --- |
| Memory | -0.60 (0.91) | 0.49 (0.53) | <.001 |
| Attention | -0.43 (0.86) | 0.34 (0.59) | <.001 |
| Executive functions | -0.55 (0.70) | 0.45 (0.38) | <.001 |
| Language | -0.63 (0.73) | 0.52 (0.54) | <.001 |
| Visuospatial functions | -0.56 (1.07) | 0.44 (0.67) | <.001 |

Values are mean (SD). All values are controlled for age, sex, and education. *P* values refer to the main effect across the groups.

**Key:** SCA, spinocerebellar ataxias; FRDA, Friedreich ataxia; HC, healthy controls.

**Supplementary Table 2: Spatial navigation data controlled for MMSE score**

|  | SCA  (n = 30) | FRDA  (n = 30) | HC  (n = 34) | *P* |
| --- | --- | --- | --- | --- |
| *Spatial navigation performance* | | | | |
| PTSOT angular deviations (degrees)^a^ | 54.43 (22.84) ***^++^ | 37.21 (21.57) | 30.93 (21.15) | <.001 |
| PTSOT correct quadrants (%)^a^ | 58.70 (21.70) *** | 62.67 (20.49)* * | 77.78 (20.09) | .001 |
| Directional-approach Task (score)^b^ | 11.78 (3.17) | N/A | 14.59 (3.14) | .001 |
| SBSOD (score)^a^ | 4.48 (1.06) | N/A | 4.74 (1.05) | .380 |

Values are mean (SD). *P* values refer to the main effect across the groups. ^a^Values are controlled for age, sex, and education, and MMSE. ^b^Values are controlled for age, sex, education, BDI, and MMSE.

Significant differences between the groups based on post-hoc analyses. ^**^p <.01, and ^***^p <.001 compared to the HC group; ^++^ p <.01 compared to the FRDA group.

**Key:** SCA, spinocerebellar ataxias; FRDA, Friedreich ataxia; HC, healthy controls; MMSE, Mini-Mental State Examination; PTSOT, Perspective Taking/Spatial Orientation Test; SBSOD, Santa Barbara Sense of Direction Scale.
